# Endogenous fluorescent reporters for heat shock proteins are not detectable after stress induction

**DOI:** 10.17912/micropub.biology.001049

**Published:** 2024-03-22

**Authors:** Priya Thirumaran, Rebecca Cornell, Roger Pocock

**Affiliations:** 1 Anatomy and Developmental Biology, Monash University, Melbourne, Victoria, Australia

## Abstract

Mitochondria and the endoplasmic reticulum (ER) utilise unique unfolded protein response (UPR) mechanisms to maintain cellular proteostasis. Heat shock proteins (HSPs) are UPR chaperones induced by specific stressors to promote protein folding. Previous research has successfully employed transgenic reporters in *Caenorhabditis elegans* to report HSP induction. However, transgenic reporters are overexpressed and only show promoter regulation and not post-transcriptional regulation. To examine endogenous HSP regulation, we attempted to generate and validate endogenous reporters for mitochondrial ( HSP-60 ) and ER ( HSP-4 ) chaperones. Using CRISPR/Cas9 technology, F2A-GFP-H2B coding DNA was inserted downstream of each HSP gene and stress induction assays conducted to validate these tools. Endogenous reporters were successfully generated for *hsp-4* and *hsp-60* . However, GFP induction could not be detected with these endogenous reporters upon stress induction, likely due to low level expression.

**Figure 1. Generation of endogenous reporters for heat shock proteins HSP-4 and HSP-60 and their validation with stress induction assays:**
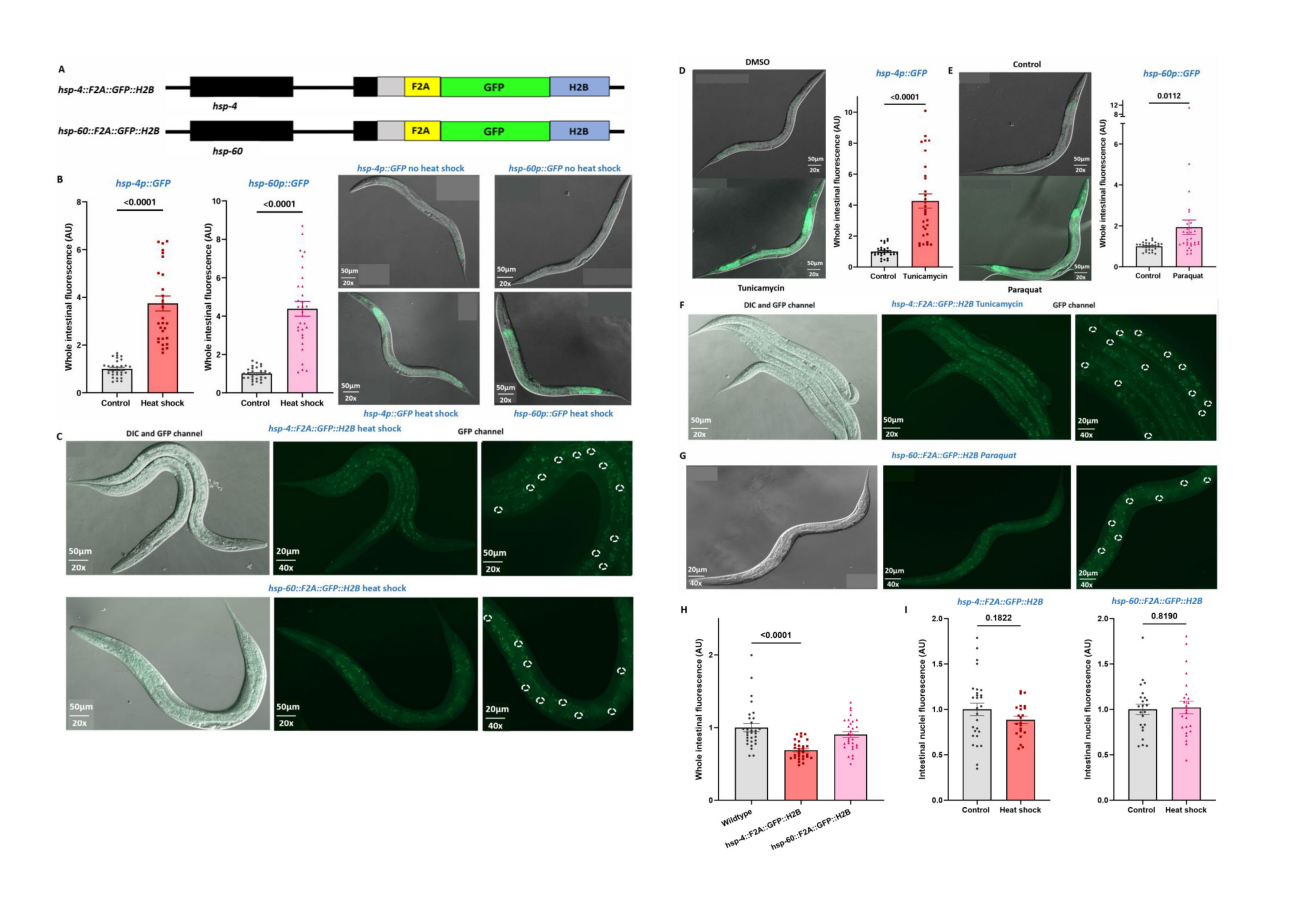
**
(A) Schematic of CRISPR/Cas9 generated *hsp-4::F2A::GFP::H2B* and *hsp-60::F2A::GFP::H2B* tools.** At the 3' end of *hsp-4* and *hsp-60* coding sequence, the following sequences were inserted using CRISPR/Cas9: a linker sequence (grey), F2A (yellow), GFP (green) and H2B (blue). **(B)** **Heat shock increased the whole intestinal fluorescence (AU = arbitrary units) of both existing *hsp-4p::GFP* and *hsp-60p::GFP* transgenic reporters.** For both tools, an approximate 4-fold increase in whole intestinal fluorescence was detected after heat shock. Representative images show an increase in fluorescence after heat shock. **(C) GFP was not visibly induced in the intestinal nuclei of *hsp-4::F2A::GFP::H2B* and *hsp-60::F2A::GFP::H2B* tools.** Representative images of both tools post-heat shock at 20x and 40x objectives show that GFP expression is not detected in intestinal nuclei (white circles). **(D)** **Tunicamycin treatment significantly increased whole intestinal fluorescence (AU = arbitrary units) in the existing *hsp-4p::GFP* transgenic reporter.** A significant increase was noted in intestinal fluorescence following tunicamycin treatment (n = 30). Representative images show the increase in fluorescence post-stress induction. **(E) Paraquat treatment significantly increased whole intestinal fluorescence in the existing *hsp-60p::GFP* transgenic reporter.** A significant increase was noted in intestinal fluorescence following paraquat treatment (n = 30). Representative images show the increase in fluorescence post-stress induction. **(F) Endogenous *hsp-4::F2A::GFP::H2B* is not visibly induced in intestinal nuclei after tunicamycin treatment.** Representative images of endogenous reporter *hsp-4::F2A::GFP::H2B* in GFP channel at 20x and 40x objectives show that no GFP expression is detected in intestinal nuclei (white circles). **(G) Endogenous *hsp-60::F2A::GFP::H2B* is not visibly induced in intestinal nuclei after paraquat treatment.** Representative images of endogenous reporter *hsp-60::F2A::GFP::H2B* in GFP channel at 20x and 40x objectives show that no GFP expression is detected in intestinal nuclei (white circles). P values were determined using unpaired Student's t-test. **(H) Fluorescence of endogenous strains is not increased compared to wild-type in unstressed conditions.** When unstressed, *hsp-4::F2A::GFP::H2B* and *hsp-60::F2A::GFP::H2B* whole intestinal fluorescence was not greater than wild-type (n = 30) suggesting that the fluorescence observed in the endogenous tools was due to background. P values were determined using one-way ANOVA. **(I) The endogenous *hsp-4::F2A::GFP::H2B* and *hsp-60::F2A::GFP::H2B* reporters are not induced by heat stress.** When exposed to heat stress (37 ^o^ C, for 2 hours), *hsp-4::F2A::GFP::H2B* and *hsp-60::F2A::GFP::H2B* nuclear fluorescence is not induced. (n = 30). P values were determined using one-way ANOVA.

## Description

Every organelle in eukaryotic cells performs distinct roles that are integral to normal cellular function. Proteostasis is crucial for maintaining organelle health and cellular homeostasis (Osellame et al., 2012; Moehle et al., 2019; Sengupta and Weljie, 2019) . Cellular stress generated by genetic variations, the environment, and dietary changes disrupt proteostasis and can lead to cell and tissue dysfunction (Fulda et al., 2010; Jiang et al., 2021) . To combat damage from toxic protein build-up, the mitochondria and ER utilise unique stress responses called the mitochondrial unfolded protein response (UPR ^mt^ ) and the ER unfolded protein response (UPR ^ER^ ), respectively (Fulda et al., 2010; Heldens et al., 2011) . Chaperone and protease genes are key players in mediating these stress responses. The primary roles of ER heat shock protein 4 ( HSP-4 ) and mitochondrial heat shock protein 60 ( HSP-60 ) chaperone proteins are to regulate protein quality control and folding to maintain homeostasis (Fulda et al., 2010) . Although the key chaperone proteins of cellular stress responses are widely known (Fulda et al., 2010; Heldens et al., 2011; Chovatiya and Medzhitov, 2014; Jiang et al., 2021; Inigo and Chandra, 2022) , there is currently no tool available that enables the *in vivo* visualization of endogenous UPR ^mt^ and UPR ^ER^ stress responses. To obtain further insight into the function and mechanics of cellular stress responses, we aimed to generate tools to analyse endogenous chaperone protein function and regulation. These tools would enable visualisation and quantification of endogenous mitochondrial and ER stress responses *in vivo* .

To investigate endogenous HSP expression, endogenous reporters for HSP-4 and HSP-60 were generated using CRISPR/Cas9 technology. The CRISPR design for *hsp-4::F2A::GFP::H2B* ( Fig. 1A, top ) and *hsp-60::F2A::GFP::H2B* ( Fig. 1A, bottom ) contained linker, F2A, GFP and H2B sequences. Both the linker sequence and F2A ribosome skipping sequence aimed to ensure that the endogenous protein function was maintained by separating the endogenous protein from the GFP fluorophore. As both HSP-4 and HSP-60 proteins are expressed ubiquitously, the histone H2B nuclear localization signal was added to direct GFP to the nuclei and thereby aid visualization. Successful in-frame insertion of the linker::F2A::GFP::H2B sequence into each gene was confirmed by Sanger sequencing prior to analysis of induction by relevant stressors.

To examine stress induction of endogenous *hsp-4::F2A::GFP::H2B* and *hsp-60::F2A::GFP::H2B* reporters we used treatments previously shown to induce the *hsp-4p::GFP* and *hsp-60p::GFP* transgenic reporters (Wallace et al., 2015) . First, we used a general heat shock treatment that induces expression of the transgenic *hsp-4p::GFP* and *hsp-60p::GFP* reporters ( Fig. 1B ). However, GFP was not visibly induced in the *hsp-4::F2A::GFP::H2B* ( Fig. 1C, top ) or *hsp-60::F2A::GFP::H2B* ( Fig. 1C, bottom ) endogenous tools following heat shock.

Next, we treated *hsp-4p::GFP* and *hsp-4::F2A::GFP::H2B* animals with the ER stress inducer tunicamycin (Guha et al., 2017) . We found that as previously described (Kim et al., 2010) , the *hsp-4p::GFP* transgenic reporter is robustly induced by tunicamycin ( Fig. 1D ). However, no induction was detected in the *hsp-4::F2A::GFP::H2B* endogenous reporter ( Fig. 1F ). Likewise, expression of *hsp-60p::GFP* is induced by the mitochondrial stressor paraquat (12) ( Fig. 1E ) but no induction was detected in the *hsp-60::F2A::GFP::H2B* endogenous reporter ( Fig. 1G ).

To confirm that any fluorescence observed in the *hsp-4::F2A::GFP::H2B* and *hsp-60::F2A::GFP::H2B* strains is due to background, we compared their fluorescence to wild-type animals when unstressed. We found that intestinal fluorescence of *hsp-4::F2A::GFP::H2B* and *hsp-60::F2A::GFP::H2B* animals was not greater than wild-type and thus the fluorescence detected was likely background ( Fig. 1H ). Similarly, there was no increase in intestinal nuclear fluorescence resulting from exposure to heat stress in the *hsp-4::F2A::GFP::H2B* and *hsp-60::F2A::GFP::H2B* strains ( Fig. 1I ).

Together, these data suggest that endogenous expression of *hsp-4* and *hsp-60* may be very low even when induced by a stressor. As the previously generated transgenic tools likely contain many promoter-GFP copies, protein expression is strongly intensified and can be visualised by fluorescence microscopy. The endogenous tools could be further validated by qPCR to detect *gfp* transcripts or western blotting to detect and quantify low-abundant proteins, however, the ability to visualize these proteins *in vivo* is at present beyond detection. Owing to the vast amount of time and resources poured into the project, we would like to share our results with the scientific community to prevent the further investment of other academics potentially attempting to generate such tools.

## Methods

Genome editing of HSPs using CRISPR/Cas9

The *Alt-R ^TM^* CRISPR HDR design tool by Integrated DNA Technologies, Inc. was used to design CRISPR sequences generated in this study. Injection mixtures for both tools were prepared according to a previous description by (Paix et al., 2015) . The components of the injection mixture, PCR primers and reaction conditions used for screening and sequencing are listed in Tables 2-4.

**Microinjection, Genotyping and Sequencing**

CRISPR-Cas9 injection mixes were injected into the germline of 25 individual adult wild-type worms (P0). Injected worms were transferred onto individual plates and incubated at 20°C for 3 days to produce F1 progeny. Each P0 plate was assessed for mCherry fluorescence. The plate with the greatest number of worms displaying the red injection marker was deemed a good candidate injection plate for screening. 200 F1 progeny were picked from candidate plates and F2 progeny were screened by PCR for the insertion. For each plate, a worm lysate was obtained, and genomic DNA used as a PCR template for detection of the insertion. Homozygosity of a candidate insertion strain was confirmed on 12 single animals. Following this, purified DNA from the sequencing PCR was collected and the in-frame inserted sequence was confirmed by Sanger sequencing.

**Stress induction assays**

All animals were maintained for three generations at 20°C prior to each stress induction assay and late L4 or early adult stage worms were primarily investigated. In each type of stress induction, transgenic *hsp-4p::GFP* and *hsp-60p::GFP* animals acted as the positive control for the assay. Animals were imaged immediately after exposure to respective stress inducers and intestinal GFP intensity was analysed. Heat shock was conducted by exposing worms to 37°C for two hours (Wallace et al., 2015) . To induce ER stress, animals were transferred onto NGM plates containing 10μg/mL tunicamycin or DMSO (control) and were incubated at 20°C for five hours prior to imaging (Kim et al., 2010) . NGM plates with tunicamycin or DMSO were prepared according to the recipe as described previously (Kim et al., 2010) . To induce mitochondrial stress, animals were transferred onto NGM plates containing 3.33M paraquat or water (control) and incubated at 20°C for five hours prior to imaging (Senchuk et al., 2017) . NGM plates with paraquat or water were prepared according to the recipe as described previously (Castello et al., 2007) .

**Microscopy**

Worms were anaesthetized with levamisole (0.1 ng/ml) for imaging expression of fluorescent reporters. Worms were mounted on 5% agarose pads on glass slides. Images were acquired using a Zeiss Axio Imager M2 and Zen software tools. Figures were prepared using ImageJ and Adobe Illustrator.

## Reagents

&nbsp;

&nbsp;

&nbsp;

&nbsp;

**Table:** 

**Reagent for**	**Component**	**Company name (RRID)**
Stress assessment	Methyl viologen dichloride hydrate (Paraquat)	Sigma Aldrich (856177)
	Tunicamycin	Sigma (T7765-1MG)

**Table 1 *C. elegans* strains used in this study**

**Table:** 

**Name**	**Genotype**
**N2 (Bristol Strain) **	Wild-type
**SJ4058**	*zcIs9 (hsp-60p::GFP) *
**SJ4005**	*zcIs4 (hsp-4p::GFP) *
RJP5900 *	*rp198 (hsp-60::F2A::GFP::H2B) *
RJP5882 *	*rp195 (hsp-4::F2A::GFP::H2B) *

*Generated in this study. Abbreviations: HSP: Heat shock proteins, F2A: ribosomal skipping sequence, GFP: Green fluorescent protein; H2B: histone 2B sequence.

**Table 2 Components of CRISPR microinjection mixture ^51^**

**Table:** 

**Component**	**Volume and Concentration**
Cas9	0.5 µl of 10 µg/µl
tracrRNA	5µl of 0.4 µg/µl
crRNA	2.8µl of 0.4 µg/µl
dsDNA donor cocktail	2.2 µl of 200 ng/µl
*Pmyo-2::mCherry* plasmid	1.6 µl of 4g/µl
Nuclease-free water (optional)	To bring total volume to 20µl

**Table 3 PCR Primers used for screening**

**Table:** 

**Gene**	**Name of Primer**	**Sequence**
***hsp-4::F2A::GFP::H2B***	Hsp-4 GFP F geno	GAATCTGTTGTTCAACCAATCG
	Hsp-4/6/60 GFP R geno	cgtcacgacttcttcaagtc
***hsp-60::F2A::GFP::H2B***	Hsp-60 GFP F geno	CCAAGATGCTTCAGGAGTCG
	Hsp-4/6/60 GFP R geno	cgtcacgacttcttcaagtc

**Abbreviations:** HSP: Heat shock proteins, GFP: Green fluorescent protein, F: Forward, R: Reverse, geno: genotype, PCR: Polymerase chain reaction.

**Table 4 PCR primers for sequencing**

**Table:** 

**Tool**	**Band size (bp), Annealing temperature (°C)**	**Name of Primer**	**Sequence**
***hsp-4::F2A::GFP::H2B***	871 bp, 58°C	RC *hsp-4* seq1 F	CAAACTTTACTCGGCGGGA
		RC *hsp-4* seq1 R	TCCGATTGGGGTGTTTTGTT
	813 bp, 58°C	RC *hsp-4* seq2 F	GGAAACATCCTCGGACACAA
		RC *hsp-4* seq2 R	GGGTTGGGTTGGGAAAGAAT
***hsp-60::F2A::GFP::H2B***	977 bp, 57.5°C	RC *hsp-60* seq1 F	CTCCAAGATGCTTCAGGAGT
		RC *hsp-60* seq1 R	TAGTGGTTGTCTGGGAGGAG
	731 bp, 58°C	RC *hsp-60* seq2 F	TCAGATCCGTCACAACATCG
		RC *hsp-60* seq2 R	AATGGCTCAGAGCACAAAAGA
